# The morpho-syntax of question particles in Standard Arabic

**DOI:** 10.1371/journal.pone.0299710

**Published:** 2024-05-24

**Authors:** Abdul-Hafeed Fakih, Ali Abbas Falah Alzubi, Sami Algouzi

**Affiliations:** Department of English, College of Languages and Translation, Najran University, Najran, Saudi Arabia; Lamar University, UNITED STATES

## Abstract

Unlike wh-question questions in Standard Arabic (SA), which received much attention in the past decades in different approaches within generative grammar, question particles (yes-no questions) in SA have not yet been studied thoroughly in minimalist syntax, and less attention has been paid to them. There is a need to analyze SA question articles and explore their syntactic behavior within minimalism. The reason why this topic has been selected for study is that SA question particles have not been investigated in detail yet in Chomsky’s Phase Theory; it has not been analyzed how question particles are derived and represented morpho-syntactically in a clause structure. Therefore, this study aims to investigate the morpho-syntax of SA question particles and provide satisfactory answers to the following questions: (i) Do question particles in SA undergo any syntactic movement to [Spec-CP] in the derivation of yes-no questions? If not, why?, (ii) Are question particles based-generated in [Spec-CP]?, and (iii) How can question particles be accounted for neatly in Chomsky’s Phase-based Theory? The paper adopts Chomsky’s Phase Theory to examine the interaction between the assumptions of this theory and the SA data on question particles. The study findings reveal that, unlike English, question particles in SA do not undergo any syntactic movement while deriving yes-no questions and are assumed to be base-generated in [Spec-CP]. Such question particles are not part of the verb morphology and are merely morphological affixes used as devices to mark interrogativity in the syntax; they do not carry any agreement and tense features that trigger syntactic movement to the clause-initial position.

## Introduction

The syntax of question particles has received considerable attention in the syntactic analyses of the world languages in the past thirty years in generative grammar, where various analyses and treatments of different syntactic approaches have been suggested and proposed in an attempt to provide a satisfactory account of the subject under investigation [1–12]. In the existing literature on yes-no questions in the world languages, much work has been observed, and very less work has been conducted on question particles in Standard Arabic (SA, henceforth). On the other hand, the wh-question movement in SA has received much attention in the past decades [13–20] where various accounts have been presented, and alternative accounts have also been suggested in different frameworks within generative grammar. However, there is no justification as to why the SA question particles have not yet been analyzed and explored in detail in minimalism. The first attempt to investigate “SA question affix analysis” was found in [21]; the analysis adopted the earlier checking framework of [4].

In this paper, two approaches are reviewed to explore research on the morpho-syntactic analysis of question particles: (i) the typological and morphological approach and (ii) the syntactic approach (in the latter approach, the pre-minimalist analyses and minimalist accounts are examined). However, neither of these two approaches discussed question particles in SA in detail (except Fakih and Al-Dera’s [21] nor could be adopted in the morpho-syntactic analysis of the topic under study.

Moreover, there are two question particles (*ʔa* and *hal*) in SA, which can be placed clause-initially to form yes-no questions. This can be demonstrated in (1) and (2).

1a. katabat hindun  risaalat-an   **VSO**

wrote Hind.nom letter-acc-indef

 ‘Hind wrote a letter.’

b. **ʔa**- katabat hindun risaalat-an   **VSO**

 **Q** wrote Hind.nom letter-acc-indef

  ‘Did Hind write a letter.’

c. **hal** katabat hindun risaalat-an   **VSO**

 **Q** wrote  Hind.nom letter-acc-indef

   ‘Did Hind write a letter.’

2a. al-walad-u  yaqra?u al-qiSSat-a   **SVO**

 the-boy.nom read  the-story-acc

  ‘The boy is reading the story?

b. **ʔa-** al-walad-u  yaqra?u al-qiSSat-a   **SVO**

 **Q** the-boy.nom read  the-story-acc

  ‘Is the boy reading the story?’

c. **hal** al-walad-u   yaqra?u al-qiSSat-a   **SVO**

 **Q** the-boy.nom read  the-story-acc

  ‘Is the boy reading the story?’

As illustrated in (1) and (2), the two question particles in SA can be used with both VSO and SVO word orders, and such particles have nothing to do with the verb tense; they are not part of the verb tense morphology. They can be used with SVO and VSO without creating any syntactic change in the verb tense or the clause structure. These question particles are morphological devices used in the sentence-initial position to derive interrogativity in SA. Moreover, (1) and (2) illustrate that, unlike English, *ʔa* and *hal* in SA are merely affixes placed clause-initially and that they have nothing to do with syntactic movement and agreement inflection, unlike the case of English auxiliary verb inversion in yes-no questions.

Given this, the current paper seeks to analyze the morpho-syntax of question particles in SA and provide a unified account of how they are formed and derived, how their syntactic behavior can be accounted for in the syntax, and how they can interact with Chomsky’s [1] Phase Theory, which is adopted as the framework for our analysis. In other words, it attempts to fill the existing research gap. Furthermore, the reason why this topic has been selected for study can be attributed to the following: (i) The morpho-syntax of question particles has not been studied thoroughly yet concerning Chomsky’s [1] Phase Theory. (ii) It has not been analyzed in detail how question particles in SA are derived and represented morpho-syntactically in a clause structure; there is a dire need to show whether or not question particles undergo movement in the syntax while deriving yes-no questions. (iii) A unified minimalist account of the treatment of question particles in SA is needed. (iv) It is important to show how SA is different from English and other languages in forming yes-no questions; English resorts to head movement/auxiliary inversion whereas SA does not have this device. Furthermore, this study aims to provide a satisfactory answer to the following research questions: (i) Do question particles in SA undergo any syntactic movement to [Spec-CP] projection while deriving yes-no questions? If not, then why?, (ii) Are question particles based-generated in [Spec-CP]?, and (iii) How can question particles be accounted for neatly in Chomsky’s [1] Phase-based Theory?

This paper is organized as follows: section 1 reviews the previous studies and surveys their morpho-syntactic accounts on question particles: two approaches are discussed (the typological and morphological approach and the syntactic approach). Section 2 introduces Chomsky’s [1] Phase Theory, which this study adopts as the framework for analyzing SA question particles. Section 3 analyzes SA question particles in SVO and VSO within Chomsky’s [1, 22] Phase Theory and, in turn, provides a unified minimalist analysis of the subject under study. Section 4 concludes with the study findings and implications.

## Literature review

This section surveys the previous analyses and accounts on question particles (termed as yes-no questions or polar questions) in some approaches in works, such as [8, 9, 11, 12, 15, 21, 23]. In this section, two approaches are discussed to examine what has been done in the morpho-syntactic analysis of question particles so far: the typological and morphological approach and the syntactic approach. In the latter approach, two sub-sections are analyzed: the pre-minimalist analyses and minimalist accounts). However, neither of these two approaches discussed question particles in SA in detail (except Fakih and Al-Dera [21] analysis that followed the earlier analysis of feature checking of [4]) nor can be adopted in the morpho-syntactic analysis of the topic under study. Moreover, some studies on the SA wh-question movement were conducted in different approaches within generative grammar, but little attention has been paid to the morpho-syntactic analysis of SA question particles. Therefore, the current study aims to study question particles (*ʔa* and *hal*) in SA within Chomsky’s [1] Phase Theory and present a unified account of the topic under investigation.

### The typological and morphological approach

Traditional and modern Arab grammarians discussed the formation of questions in SA within their traditionally taxonomic approach to grammar and provided a brief description of the typological and morphological analysis of yes-no questions. Their accounts reflected disagreement with each other on the syntactic analysis of the question particles, *ʔa* and *hal*, on the one hand, and the question words on the other. For instance, some Arab grammarians such as [24–27], and some modern Arab grammarians, such as [28, 29] tended to use the terminology *ħuru*:*fu al-*?*istifhām* "particles of interrogation" to mean both the question particles and question words/phrases, thus confusing the difference between question particles and wh-words [15, 21]. Moreover, their descriptive analyses could not provide a convincing distinction between these two different types of question formation, and whether or not the question particle or wh-word moves to the clause-initial position.

Holmberg [10] discussed polar questions in languages that have VO word order and demonstrated that these languages pose a challenge for (what he dubbed as) Final-Over-Final Constraint (FOFC) that rules out a head-final phrase, which immediately dominates a head-initial phrase. Furthermore, Bailey [8] provided a typological description of question particles and supported Holmberg’s hypothesis that final question particles differ fundamentally from their initial counterparts. Bailey explored the syntax of question particles, which combine with a declarative clause to form a yes-no question and indicated that some of such question particles violated the principle (FOFC, assumed by Holmberg) to be universally valid. Bailey’s concern was how languages with VO order and sentence-final question particles violate FOFC. Bailey indicated that some analyses of question particles were analyzed and then were rejected because they were found ‘impossible to maintain concurrently with FOFC.’ This entails that question particles are problematic for FOFC.

Bailey [9] stressed that polar questions can be derived in different ways cross-linguistically. For instance, in English polar questions are formed by changing the clause word order, where a tensed auxiliary is placed sentence-initially (for example, *Does Mary play tennis every day*? Bailey observed that word order change strategy in English is extremely rare in the world languages; only a few languages do form questions in this strategy, this can be found in some Indo-European languages: Czech, Danish, Dutch, English, Frisian, German, Norwegian, Spanish and Swedish. Bailey examined polar question particles in Thai and Japanese and indicated that in both languages, the polar question particles *máy* and *ka*, respectively, are used in the sentence-final position. Both languages illustrate some similarity in the derivation of question-forming strategy; however, some differences surface concerning the type of question in which the particle can occur in the syntax.

Dryer [23] presented a map (dubbed Map 116) that shows the position of question particles in polar questions in the world languages, as illustrated in Table 1:

**Table 1:** Map of the position of question particles in polar questions.

	Question particles position	Number
1	Question particle at beginning of sentence	118
2	Question particle at end of sentence	272
3	Question particle in second position in sentence	45
4	Question particle with other position	8
5	Question particle in either of two positions	24
6	No question particle	311
	TOTAL	777

Dyer [23] demonstrated that “Map 116” illustrates the distribution of different strategies employed by world languages to form polar questions, including question particles. It discusses various ways in which a question particle can occur in the interrogative (whether in the beginning, second position, end, with other position, either of two positions, or no question particle in the sentence).

Moreover, Holmberg [11] described Finnish yes-no questions as formed by adjoining the enclitic particle– *ko* to the first constituent of the interrogative clause. The enclitic particle *ko* can be realized as *-ko* or *-kö* and is also subject to vowel harmony while deriving the interrogative structure in the syntax. This can be illustrated in (3).

3a. Ajoi-ko Olli illalla kaupunkiin?  **Finnish**

 drove-WH Olli evening-ADE town-ILL

 ‘Did Olli drive into town in the evening?’

b. On-ko kukaan muistanut tuoda sokeria?

 has-WH anyone remembered bring sugar

 ‘Has anyone remembered to bring sugar?’  [11, p.1]

Holmberg is not with the view that the question particle in Finnish is used in the second position of the interrogative sentence as a clitic and that it is derived by merging it as a species of the head C in the clause left periphery, and moving a constituent to the Spec-CP configuration. Holmberg indicated that this line of analysis is not a viable account of the question particle in Finnish. Holmberg discussed two alternative theories and found that neither of them takes the Finnish enclitic question particle *–ko* as a C element. Like –*kin*, Holmberg noted that *-ko* has a focus feature which is unvalued, and which specifies its distribution concerning focus.

Holmberg’s [11] account of the question particles in Finnish is a mixture of a morphological and typological analysis, which is not based on Chomsky’s phase-based analysis. However, the researchers do not adopt this approach in their analysis of the SA question particles. They adopt Chomsky’s [1, 30] Phase Theory, which is more economical and can provide a unified account of the topic under investigation.

Romero and Meertens [31] explored the syntax of the Q-particle *də* in Sinhala wh-questions and polar questions and indicated that the Q-question particle in Sinhala *də* obligatorily appears in wh-questions (WhQs), polar questions (PolQs) and alternative questions (AltQs). Cable [12] and Romero and Meertens [31] stressed that the Q-particle *də* is syntactically the head of a Q-particle phrase (QP). In addition, Bhatt and Dayal [32] examined the polar *kya*: in Hindi and Urdu and made a distinction between two types of interrogative particles: one type of interrogative particle is typically referred to as a Q-morpheme in the existing literature; this is viewed to be the overt realization of C[+Q]. The other one was treated as a polar question particle (PQP), which can be used only in a subset of clause-types marked C[+Q]. That is, the first can be used in all interrogatives, while the second polar particle *kya*: can occur in polar questions but not in wh-questions.

### The syntactic approach

#### Pre-minimalist analyses

The first modern syntactic analysis of question formation was initiated in Chomsky’s [2] *Syntactic Structures*, where he departed from the structural paradigm to the generative-transformational paradigm. In deriving yes-no questions in English, Chomsky [2] posited the following underlying structure in (4).


4John‐C‐eat+an+apple(NP–C‐V…)

Chomsky noted that the dashes in (1) demonstrate that the syntactic analysis is imposed by a transformational rule notated as Tq; this Tq rule is assumed by Chomsky to explain the derivation of yes-no questions in English. Chomsky proposed two transformational (optional and obligatory) rules to show how yes-no questions could be formed and pointed out that yes-no questions could be derived utilizing a transformation Tq that operates on strings. On the other hand, Katz and Postal [33] disagreed with Chomsky’s [2] treatment of questions: the latter demonstrated that a question and its corresponding declarative have the same sequence of underlying P-marker(s), and yet they differ in meaning. Katz and Postal [33] argued that questions are not genuine counterexamples as they are derived from structures containing Q-morpheme. The Q-morpheme hypothesis owes its origin to Katz and Postal’s [33] analysis. However, Baker [34] argued against Katz and Postal’s [33] abstract Q-morpheme proposal and noted that English direct and indirect questions have an initial Q-morpheme in the deep structure. Other arguments that were put forward against the Q-assumption were discussed in the works by [35–39]. Moreover, in the *Barriers* of the GB approach, Chomsky [3] modified the clause structure and added the CP projection (above IP projection) in order to host yes/no questions in the head C position and wh-words in the specifier of CP.

#### Minimalist accounts

In the Minimalist Program, Chomsky [4] departed from the earlier treatment of the Q-morpheme hypothesis and proposed the feature-checking approach based on ‘economy principles.’ Chomsky explored the abstract question affix Q (= [+wh]) and assumed its presence in the deep structure of the interrogative structure. Chomsky proposed that the complementizer C in an interrogative clause contains an abstract question affix Q, with a strong Q-feature, as demonstrated in (5).

5 Q[IP John gave DP to Mary] [4, p.289]

On the other hand, Chomsky indicated that as languages differ in the strength of the Q-feature, “the strong Q-feature is satisfied by a feature FQ”, [4, p.289]. The abstract affix Q is strong in English and that in the process of feature checking, the Q-feature must be eliminated in the syntax ‘by insertion of FQ’ in its licensing domain; once FQ is checked in the domain, it erases Q by Merge or Move, by substitution or adjunction.

Fakih [15, 21] explored question affix analysis (?*a* and *hal*) in SA within Chomsky’s [4] feature-checking approach. Fakih and Al-Dera [21] demonstrated that SA differs from English in terms of feature strength, feature checking, and I-raising to Q (i.e., the raising of the head INFL to the head COMP). Fakih indicated that the yes-no question formation device is not the same in these two languages; SA and English differ significantly in the formation of yes-no questions in the syntax. Unlike SA, a yes-no question in English demands fronting a tensed auxiliary/modal verb or a dummy *do* in the initial position of the interrogative; that tensed auxiliary must be moved to the clause-initial position, thus forming a question. This cannot be the case in SA, which lacks the tensed auxiliary device. This can be illustrated in (6).

6 **ʔa** – ħadara  zayd-un  al–mu?tamar–a?

  **Q** attend-pst-m.sg. Zaid-nom. def-conference -acc.

  ’Did Zaid attend the conference?’   [21, p.10]

Fakih’s analysis is seen to be the first attempt to explore question affix analysis in SA. However, it followed the earlier version of the Checking Theory of Chomsky’s [4] Minimalist Program. Recent developments in syntactic theory have shown that the current Phase Theory of Chomsky [1] can provide a straightforward analysis of syntactic phenomena in languages. Therefore, the present study adopts the Phase Theory proposed by Chomsky [1, 30], the latest theory in minimalism, to account for the morpho-syntax of question particles in SA.

Moreover, Radford [5, 6] discussed auxiliary inversion in English yes-no questions involving an I-movement, where an auxiliary verb moves up from INFL to COMP. Radford indicated that Q must be affixed either to an interrogative complementizer or an auxiliary and noted that an interrogative COMP is strong in English and, hence, can motivate the movement of an auxiliary from INFL to COMP. However, Radford left unanswered the question of whether a strong interrogative COMP can or cannot contain an affix Q. This entails that Radford did not support the claim that there is an abstract question affix Q in the head C of an interrogative clause. In other words, Radford disagreed with Chomsky’s assumption of the presence of Q in the head C position of an interrogative. Radford discussed a puzzling phenomenon of yes-no question formation in English, where a yes-no question can be derived from a clause having no auxiliary verb, thus requiring the use of the dummy *do* (known as do-support), as shown in (7).

7a. They know him.

b. Do they know him?  [5, p.109]

Radford suggested that the interrogative head COMP of CP is strong in today’s Modern English and has to be filled, while the head INFL of IP is weak, and need not be filled. The dummy not only fills COMP but also undergoes movement from INFL to COMP, thus leaving behind a trace, which Radford terms as ‘a silent copy of itself’. Furthermore, Radford [6] stated that the operation Move has two sub-operations: Copy and Delete. To illustrate this in a yes-no question such as *Can John play tennis*? the syntactic derivation of this interrogative begins by merging the main verb *play* with the object NP *tennis* to form the VP *play tennis*. Next, the VP, *play tennis*, is merged with the tensed auxiliary *can* to derive the T`*can play tennis*. Then, T`, *can play tennis*, is merged with the object NP *tennis* to form the TP projection *John can play tennis*. Consequently, the TP, *John can play tennis*, is merged with the head C, which bears the Question feature [Q], to form the CP projection. Radford stressed that a copy of *can* moves and merges with the head C under head movement, resulting in a complex C, since it carries a copy of T, *can*, and the feature question [Q]. Radford [6] indicated that a copy of the moved T stays in-situ, whereas its phonetic feature will be deleted in the phonological component of the syntax.

On the other hand, in his analysis of the grammar of Q, Cable [12] argued that there is no syntactic relationship between the interrogative C and the wh-phrase involved in wh-fronting in the Tlingit language. However, Cable stated that there is a ‘probe/Agree’ relation between the head C of CP and an overt Q-particle that c-commands the wh-phrase and that the wh-phrase movement in Tlingit wh-questions is viewed as a mere by-product of moving the projection of the Q-particle. Cable pointed out that the wh-phrase should be c-commanded by the Q-particle *sa*; such a Q-particle heads its projection, (dubbed as ’QP’). However, this line of analysis cannot apply to SA because the question particles *ʔa* and *hal* cannot be analyzed in Cable’s [12] framework. The SA question particles can be analyzed independently of wh-questions. SA question particles have nothing to do with wh-phrases, and both cannot be used in the same interrogative structure; they are in complementary distribution. This issue will be discussed in the section on SA question particles. For Chomsky [4, p, 289], "FQ is often called the wh-feature."

### Strategies of deriving yes-no questions

There are many ways of deriving yes-no questions in languages. Let us introduce the most common single question-marking device employed by human languages. It has been observed that in the majority of languages, the question particle appears in the first position of the clause [8]. The first and most common way of forming yes-no questions is by using a particle, as illustrated in (8).

8a. **czy** Marta lubi koty?   **Polish**

 **Q** Martha like.3SG cat.ACC.PL

  ‘Does Martha like cats?’  [8, p.24]

b. **la** k’ol Aa Teeko chjaay?   **Tzotzil, Mexico**

 **Q** be youth Diego at.home

  ‘Is Diego at home?’   [40, p.330]

c. **ʔa–**  ħadara zayd-un al–mu?tamar–a?   **Standard Arabic**

 **Q**  attend-pst-m.sg. Zaid-nom. def-conference -acc.

  ’Did Zaid attend the conference?’   [21, p.10]

The second common interrogative structure is using verbal morphology employed by languages, which are overwhelmingly OV in nature, such as Uzbek, Korean, and West Greenlandic. Interrogative verbal morphology was viewed by Dryer [23] as an affix that signals that the utterance is an interrogative, as demonstrated in the Tunica language example in (9):

9 lɔ’ta wi-wa’na **-n   Tunica (isolate, Mississippi)**

 run  2SG-want-**Q**

 ‘Do you want to run?’   [41, p. 118]

The third type demonstrates that in some languages the question particle appears in the second position in the interrogative sentence, as in (10) and (11).

10 me-**ne**  fugis?   **Latin**

 1SG.ACC-**Q** flee.2SG

  ‘Is it me you are running away from?’ [8, p.26]

11 sataa-**ko** ulkona?  **Finnish**

 rains-**Q** outside

  ‘Is it raining?’  [8, p.26]

The fourth type illustrates that the question particle in some other languages, on the other hand, appears in the final position. This is very common in Japanese, as shown in (12).

12 Taroo-ga hon-o  kaimasita  **ka**?   **Japanese**

 Taroo-NOM book-ACC bought.POL **Q**

  ‘Did Taro buy a book?’   [42, p.5]

The fifth type is the strategy of using a special word order (with a tensed auxiliary, which moves to the clause-initial position to derive an interrogative), as the case in English, as shown in (13). English also uses tag questions and intonation to form yes-no questions.

13a. John is reading a book.

b. Is John reading a book?

c. Mary played teniis yesterday.

d. Did Mary play tennis yeserday?

f. She is washing the dishes, isn’t she?

The sixth type is the use of interrogative intonation, assumed to be present in some languages whose normal question intonation is a final rising contour [8, 10]. This can be found in languages, such as Maori (New Zealand) and Kikuyu (Kenya).

### Summary

The preceding section discussed how the typological and morphological approach and the syntactic approach treated question particles in different languages within the generative paradigm. However, neither of these approaches explored question particles within Chomsky’s [1] Phase Theory. Moreover, the present study does not adopt any of the preceding approaches in the formal treatment of question particles in SA and consequently adopts a different framework based on Chomsky’s [1] Phase Theory because it is straightforward and more economical; it can help answer the study questions, fill this research gap, and provide a satisfactory account of the topic under investigation.

### Introducing Chomsky’s [1, 22, 30] Phase Theory

This section seeks to introduce Chomsky’s [1, 30] Phase Theory in a simplified manner. A closer look at Chomsky’s Phase Theory reveals that it is the latest empirical and conceptual shift in minimalist syntactic theory within the Chomskyan generative paradigm. The term ‘phases’ owns its origin for the first time in Chomsky’s [22] Minimalist Inquiries; Chomsky introduced phases (as lexical subarrays associated with phases) to be a kind of solution to a challenge resulting from the syntactic analysis of the Merge over Move (MOM) principle. Moreover, the notion of a phase was built upon many significant principles, including locality domains, such as cycles, barriers, islands, etc. [43].

Furthermore, in examining the phasehood properties, Chomsky [22] viewed phases as ‘natural syntactic objects,’ ‘relatively independent in terms of interface properties’ (p.106). The central idea is that the conceptual-intentional (CI) interface demands phases to be complete from a semantic angle. Chomsky [22] stressed, “a phase is the closest syntactic counterpart to a proposition: either a verb phrase in which all theta roles are assigned or a full clause including tense and force” (p. 106). Given this, Chomsky proposed that CPs and v*Ps are phases. For Chomsky [1], transitive and unergative vPs are phases, but TPs, unaccusative and passive vPs are not. The difference between verb phrases that are not phases from those that are can be attributed to the fact that the former (verb phrases) lack external arguments. Chomsky [1, 30] indicated that the functional categories CP and v*P are phases and have their functional heads: C and v, respectively. These functional heads are more powerful than non-phase heads, and they have their properties; this stems from the fact that they are the loci of uninterpretable features that must be valued while deriving the required structure, and, consequently, they trigger syntactic operations in minimalist syntax. Chomsky [1, 22] assumed that the phase heads trigger syntactic operations and are also subject to strong cyclicity. Such functional phase heads are viewed as syntactic engines of every morpho-syntactic derivation aimed at deriving a grammatical structure. Chomsky [22] mentioned that “The head of a phase is inert after the phase is completed, triggering no further operations (p. 107).

**Fig 1:**
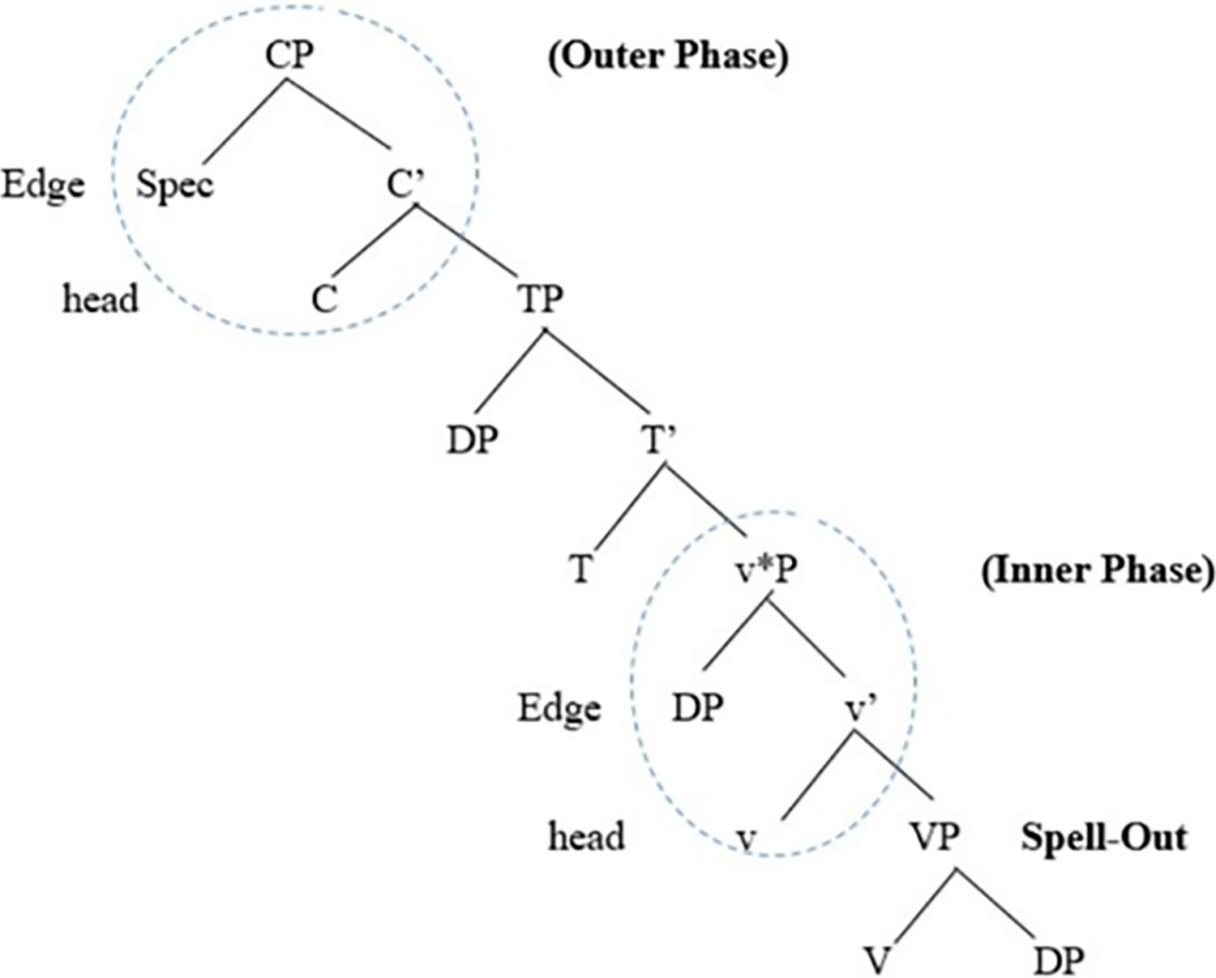
Representation of Chomsky’s Phase Theory in a clause structure.

Let us demonstrate how Chomsky’s Phase Theory operates in the syntax and how it is represented in a clause structure, as shown in Fig 1.

The clause structure in Fig 1 illustrates an inner VP and an outer v*P shell. It also shows that root clauses are introduced by CPs to indicate whether the clause represents an imperative, interrogative, exclamative, or declarative force. Moreover, Chomsky [1, 22, 30] viewed CP and transitive v*P as propositional phases. Chomsky’s motive behind assuming CP and v*P to be phased stems from the assumption that CP represents a complete complex (that has force marker: indicative, interrogative) and v*P represents a complete thematic complex that has an external argument NP. Chomsky stressed that CP and v*P are functional categories and have their functional heads. That is, C and v are phase heads, and there are syntactic operations that show an agreement relation between a probe and a local goal. Moreover, the functional heads C, T, and v are probes, and merger operations apply before the probing strategy takes place in the syntax. Chomsky [1] suggested that TP (dominated by and being within CP) is a complete clause while infinitival embedded clauses (that lack CPs) are assumed to be defective TP clauses. On the other hand, Chomsky mentioned that defective TPs and vPs lacking external thematic argument NPs cannot be phases. Concerning probing operations, Chomsky indicated that probes can search for goals either simultaneously or sequentially. Besides, it is assumed that when all syntactic operations have been completed in a particular phase, the complement of the phase head becomes impenetrable to any further syntactic operations; this is what Chomsky [30] termed the Phase Impenetrability Condition (PIC). Chomsky explained that the reason why the complement NP or the domain of the phase becomes impenetrable can be attributed to the fact that the domain undergoes transfer simultaneously to the phonological interface and the semantic component is assigned the appropriate representations in the syntax. Chomsky [22, p. 108] defines PIC as “In phase α with head H, the domain of H is not accessible to operations outside α; only H and its edge are accessible to such operations.”

### Question particles in SA

#### An overview of SA

SA is the official language of more than 20 Arab countries in the Middle East and North Africa and is spoken by hundreds of millions of people across the Arab world. It is a branch of the Semitic language family. It is classified as a Central Semitic Language. It is the language of the Holy Qur’an, the Prophet Mohammed’s sayings (Hadiths), and literature. It has a rich inflectional and derivational morphology, a rich agreement inflection, and an overt case-marking system. SA is conventionally written with the Arabic alphabet from right to left. Nouns in SA have three syntactic cases: nominative, accusative, and genitive; the genitive case is also used when it is governed by a preposition. It exhibits three numbers (singular, dual, and plural), has two genders (masculine and feminine), and possesses three "states": indefinite, definite, and construct. What is fascinating in SA is that, like nouns, adjectives are marked for case, gender, number, and definiteness. Moreover, pronouns are marked for number, gender, and person; they are either independent or enclitic; the latter is attached to the end of nouns, verbs, or prepositions and illustrates verbal and prepositional objects or possession of nouns. Furthermore, SA verbs are also marked for number, gender, and person (first, second, or third).

Moreover, it has been shown in the syntactic analyses of traditional Arab grammarians such as [24–27] and modern Arab grammarians such as [29] and [28] that Standard Arabic exhibits two prominent word orders: SVO and VSO. Modern Arab syntacticians such as [13–15, 17–21, 44, 45] stress that VSO is the unmarked word order while SVO is the marked order. Some of the Arab modern syntacticians stress that SVO is the basic word order in Standard Arabic, where the subject DP is generated in the position of the specifier position of VP. On the other hand, VSO results from a verb raising in the sense of [13, 44, 45], among others. That is, VSO is derived by the overt movement of the verb to the tense head position of TP and then to the C head of CP for feature valuation, while the subject DP remains in situ in the syntax [14, 15, 21, 44]. Some other scholars argue that SVO is the deep stricture in SA while VSO is the surface structure. Furthermore, in the analysis of SVO and VSO word orders, we show that the question particles *ʔa* and *hal* can be placed in the clause initial position of yes-no questions in Standard Arabic. That is, it does not make any difference whether the order is VSO or SVO because the question particle is placed clause -initially in the derivation of a yes-no interrogative.

In VSO order, there is a partial agreement between the verb and the subject in gender, while there is a full agreement (number, person, and gender) between the subject and the verb in SVO, as illustrated in the following examples (14) and (15).

14a? akal-at al-bint-u al-tuffaahat-a   **VSO**

ate-F-Sg def-girl-nom def-apple-acc

‘The girl ate the apple.’

b.? akal-at al-bintaani al-tuffaahat- a   **VSO**

ate-F-Sl def-girls-dual-nom def-apple-acc

‘The two girls ate the apple.’   ***(Dual)***

c.? akal-at al-banaat-u al-tuffaahat- a   **VSO**

ate-F-Pl def-girls-Pl-nom def-apple-acc

‘The girls ate the apple.’

15a. al-walad-u? akala al-tuffaahat-a   **SVO**

def-boy-nom ate-M-Sg def-apple-acc

‘The boy ate the apple.’

b. al-waladaani? akal-aa al-tuffaahat-a   **SVO**

def-boy-nom ate-M-dual def-apple-acc

‘The two boys ate the apple.’   ***(Dual)***

c. al-?awlaad-u? akal-uu al-tuffaahat-a   **SVO**

def-boy-Pl-nom ate-M-Pl def-apple-acc

‘The boys ate the apple.’

The examples in (14) show a partial agreement between the verb and the subject NP in gender in the VSO order whereas SVO examples in (15) exhibit a full agreement between the subject NP and the verb in the three phi-features (number, person, and gender). In VSO, there is a default agreement between the vert and the subject. Overt case marking is observed in (14) and (15); nominative subject DPs are marked with *-u* while accusative object DPs are marked with *-a*. So, the nominative case marker is *-u* whereas the accusative case marker is *-a*. Furthermore, definiteness in SA can be realized in (14) and (15) via the definite marker *al-* ‘the,’ which is prefixed to the subject and object DPs.

#### Analysis of question particles in SA

This section seeks to analyze the morpho-syntax of the question particles *ʔa* and *hal* in SA and present a novel analysis. We adopt Chomsky’s [1, 22, 30] Phase Theory as our approach to the morpho-syntactic analysis of question particles in SA. The paper’s objective is to provide suitable answers to the following questions: (i) Do question particles in SA undergo any syntactic movement to [Spec, CP]? If not, then why? (ii) Are SA question particles based-generated in [Spec, CP]? and (iii) How can such question particles be accounted for neatly within Chomsky’s [1] Phase-based Theory? The following sub-sections discuss the question particles *ʔa* and *hal* in deriving yes-no questions in SVO and VSO word orders.

### Derivation of yes-no questions in SVO structures

There are two question particles in SA grammar: *ʔa* and *hal*, which can be used in the clause-initial position of a sentence to form an interrogative structure. This sub-section aims to discuss the use of *ʔa* and *hal* in the SVO word order. Let us consider the following examples (16) and (17) to illustrate the point.

16a. **ʔa-**mohahammad-un darrasa al-Tullaab-a

 **Q** Mohammed-nom taught the-students-acc

 ‘Did Mohammed teach the students?’

b. *mohammad-un **ʔa-**darrasa al-Tullaab-a

 Mohammed-nom **Q** taught the-students-acc

 ‘Did Mohammed teach the students?’

**c. ʔa-**mohammad-un  yudarrisu al-Tullaab-a

 **Q** Mohammed-nom teaches the-students-acc

17a. **hal** mohammad-un darrasa al-Tullaab-a

 **Q** Mohammed-nom taught the-students-acc

 ‘Did Mohammed teach the students?’

b**. hal** mohammad-un  yudarrisu al-Tullaab-a

 **Q** Mohammed-nom teaches the-students-acc

 ‘Does Mohammed teach the students?’

c. *mohammad-un **hal**  yudarrisu al-Tullab-a

 Mohammed-nom **Q** teaches the-students-acc

 A closer look at the examples in (16) and (17) reveals that, unlike English, the SA question particles *ʔa* and *hal* are not part of the tense of the verb morphology and do not undergo any syntactic movement; they are merely morphological affixes that must be placed in front of the first constituent of the interrogative construction to mark interrogativity. This can explain why (16b) and (17c) are not acceptable in SA grammar; their ungrammaticality stems from the fact that the question particles must not be placed clause-medially or clause-finally. Such question particles must always be used in the clause-initial position. Furthermore, (16) and (17) show that these two question particles can be used with any verb tense; they are not affected by the tense (whether it is present or past). *ʔa* and *hal* are used with verbs in the past tense as in (16a) and (17a) and with verbs in the present tense as in (16c) and (17b) without changing their morpho-syntactic shape. In other words, the question particles in (16) and (17) are expected to show agreement with verbs and their subject DPs but they do not reflect that; they do not demonstrate any grammatical agreement in terms of the three phi-features (i.e., person, gender and number). It can be made clear that *ʔa* and *hal* have nothing to do with agreement inflection shown on the verbs and their subject DPs. Besides, such question particles do not inflect for tense or Case. This is different from the English tensed auxiliary verbs used to form yes-no questions; the auxiliary is part of the verb tense morphology in English [4, 6]. The auxiliary verb in English undergoes syntactic movement to the head C of [Spec, CP] for feature valuation considerations.

 The bottom line is that the question particles *ʔa* and *hal* do not carry tense and agreement features. Hense, such particles are not part of the verb morphology and do not undergo any syntcatic movement. The evidence is that any moved constituent in the clause must leave an empty category (i.e., trace) and that, while moving, the moved entity must have the right landing site [4, 46], but this does not work well with the syntactic movement of these two question particles in (16) and (17). Therefore, such question particles are treated as two interrogative morphemes, where *ʔa* is a bound morpheme and *hal* is a free morpheme; both must be placed clause-initially to mark interrogativity. Moreover, it can be observed that *ʔa* is always used as a bound morpheme, while *hal* is always used as a free morpheme, as shown in (16) and (17) above. In other words, *ʔa* cannot be used as a free morpheme and neither *hal* as a bound morpheme, as shown in the ungrammatical interrogatives in (18a-b) below.

18a. *hal—mohahammad-un darrasa al-Tullab-a

 **Q** Mohammed-nom taught the-students-acc

b. *ʔa- mohahammad-un darrasa al-Tullab-a

 **Q** Mohammed-nom taught the-students-acc

c. **hal** mohahammad-un darrasa al-Tullab-a

 **Q** Mohammed-nom taught the-students-acc

d. **ʔa-** mohahammad-un darrasa al-Tullab-a

 **Q** Mohammed-nom taught the-students-acc

 (18c) shows that *hal* must always be used as a free morpheme, while *ʔa* in (18d) must always be attached to the first constituent of the interrogative; the reverse is not acceptable and leads to ungrammaticality, as shown in (18a) and (18b).

 On the other hand, the question particles *ʔa* and *hal* cannot be used with wh-phrases in the same interrogative structure, as the case in Cable’s [12] analysis of Tlingit language. That is, *ʔa* and *hal* are in complementary distribution concerning wh-questions. This can be demonstrated in (19) below.

19a. *man ʔa-/ hal kataba qiSSat-an

 **who Q** wrote story-acc-indef

b. *maathaa shaahada **ʔa-/ hal** al-walad-u

 **what** saw **Q** the-boy-nom

The ungrammaticality of (19a-b) can be attributed to the fact that SA grammar does not permit using question particles with wh-phrases in the same interrogative structures. This is ruled out in SA syntax. Here, SA differs from other languages, such as Tlingit discussed in Cable [12] and Sinhala explored in [31] in the section on literature review.

On the other hand, the questions that arise are: Can the question particles *ʔa* and *hal* be used with negation? and Is there any difference between these two question articles while deriving a negative yes-no question? Let us explain how such question particles can interact with negation in the following examples (20).

20a. **hal/ ʔa**-waSala mohammed-un al-maTaar-a mubakkiran

 **Q** arrived Moahmmed-nom the-airport-acc early

 ‘Did Mohammed arrive at the airport early?’

b. **ʔa-maa** waSala mohammed-un al-maTaar-a mubakkiran

 **Q not** arrived Moahmmed-nom the-airport-acc early

 ‘Did Mohammed arrive at the airport early?’

c. *hal maa waSala mohammed-un al-maTaar-a mubakkiran

 **Q not** arrived Moahmmed-nom the-airport-acc early

d. **ʔa-lam** yaSel Mohammed al-maTaar-a mubakkiran

 **Q not** arrived Moahmmed-nom the-airport-acc early

 ‘Did Mohammed arrive at the airport early?’

e. *hal lam yaSel Mohammed al-maTaar-a mubakkiran

 **Q not** arrived Moahmmed-nom the-airport-acc early

A closer look at the derivation of yes-no questions in (20) reveals that the question particle *ʔa* can be used with negative yes-no questions, as shown in (20b) and (20d), while its counterpart *hal* cannot, as demonstrated in the ungrammatical interrogatives in (20c) and (20e). The similarity between *ʔa* and *hal* is that both of them are used with affirmative yes-no questions, as illustrated in (20a). However, the difference between *ʔa* and *hal* is that the former (*ʔa*) is used with both affirmative and negative yes-no questions, whereas *hal* is only used with affirmative interrogatives. *ʔa* is used to inquire about the content of the affirmative and negative yes-no questions. Furthermore, it can be observed that the unacceptability of (20c) and (20e) is due to the incorrect insertion of the negative particles *maa* and *lam* (that means *not* in a yes-no question) with the question particle *hal*. It is obvious that there is a selectional restriction about the use of *hal* in SA yes-no interrogatives; *hal* is banned from being used in negative yes-no interrogatives.

A closer examination at the syntactic differences between the polar question particles *ʔa* and *hal* reveals that *ʔa* is used in both affirmative and negative yes-no questions, while *hal* is used only in affirmative interrogatives. For syntactic suitability, let us use the term ‘affirmative’ instead of the term ‘positive’: The former (i.e., ‘affirmative’) is more formal and spells out the syntactic differences more than the latter. In order to distinguish between these two polar interrogative particles, they can be notated with plus/ minus features as follows: *ʔa-* [+Q, ±affirmative] and *hal* [+Q, +affirmative].

Furthermore, it can be observed that the negative marker in SA is a free morpheme (*maa*, *lam*, *laysa* ‘not’), not a bound morpheme, as illustrated in (20). That is, the negative marker cannot be used as an attached form affixed to constituents, but rather as a free entity. We have already pointed out that the question particle *ʔa* is always used as a bound morpheme, whereas *hal* a free morpheme. The question arises here is: why can *hal* not be used with negative yes-no questions, while its counterpart *ʔa* can? The answer can be assumed that while deriving a negative yes-no question in SA, no two free morphemes (i.e., *hal* + negative marker) can be used together in an interrogative, where one follows the other, as shown in the ill-formed constructions in (20c) and (20e) above. It can also be assumed that a bound morpheme such as, *ʔa* can precede a free negative marker (such as, *maa*, *lam* ‘not’) in the same structure. That is, the construction (a bound morpheme *ʔa* + a negative free marker) can co-occur in the same interrogative in SA. It can be concluded that the syntactic representation: *hal + neg … is not allowed, while *ʔa* + neg … is licensed in SA grammar. Any violation of this syntactic representation will lead to ill-formedness, as demonstrated in (20c) and (20e) above.

Let us now examine how the SA question particles can interact with Chomsky’s [1, 22, 30] Phase Theory and how yes-no questions can be derived and represented in the syntax. This can be demonstrated in (21).

21a. al-rajul-u banaa madrasat-an

 the-man-nom built school-acc-indef

 ‘The man built a school.’

b. **ʔa/ hal** al-rajul-u banaa madrasat-an

 **Q** the-man-nom built school-acc-indef

**Fig 2:**
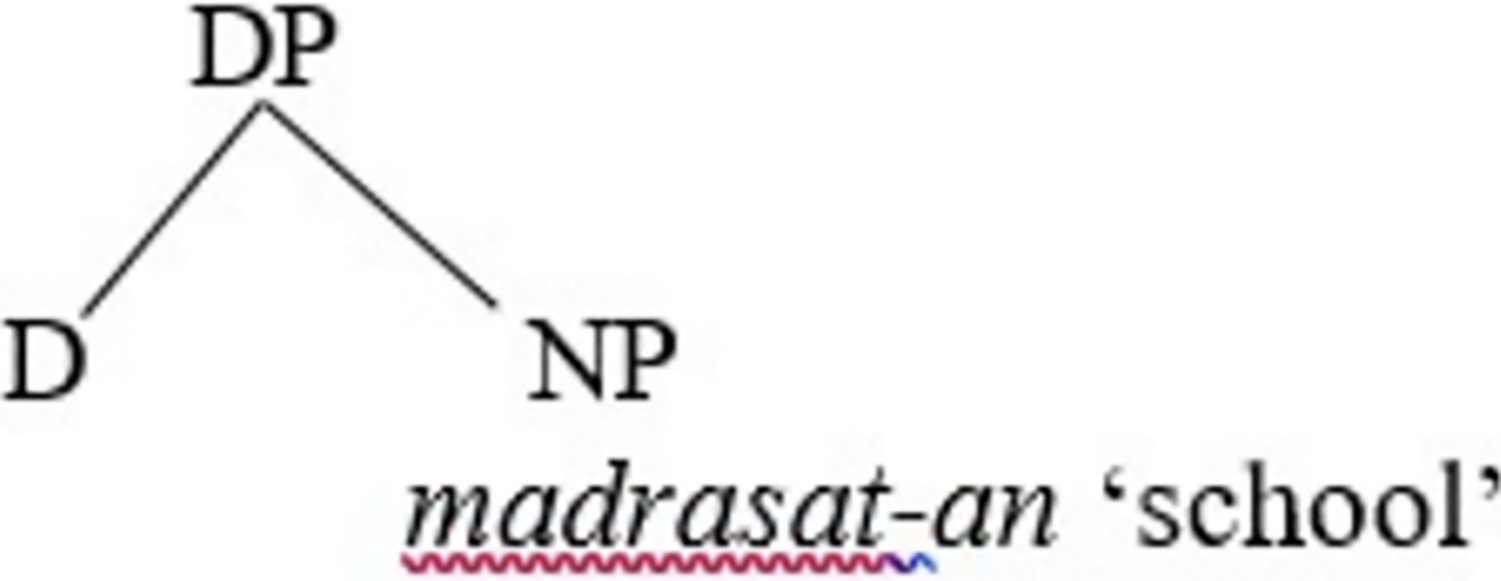
Merge of the lexical object DP *madrasat* with an indefinite determiner (*-n*) in SA.

 ‘Did the man build a school?’

(21a) is a declarative sentence, while (21b) is a derived yes-no question from (21a). It can be observed in (21b) that *ʔa* and *hal* can interchangeably be used with an affirmative interrogative. Let us assume that the subject NP *al-rajul-u* ‘the man’ is base-generated in the [Spec, vP] projection, following [47], and which then moves to [Spec, TP] for EPP-feature valuation considerations. Let us explore how (21b) is derived and how it proceeds further in the syntax. First, the lexical object NP *madrasata* ‘school’ merges with an indefinite determiner (*-n*) to form the DP *madrasatan* ‘school’ that carries a [+N] feature and unvalued accusative case feature. In SA, the definite marker is *al-* ‘the,’ while the indefinite marker is the nunation *-n* suffixed to nouns, as shown in *madrasatan* ‘a school’; *madrasat* is the root and the suffix *-a* (that follows it) is the accusative case marker, while the final suffix *-n* is the nunation, which shows indefiniteness. This can be illustrated in Fig 2.

**Fig 3:**
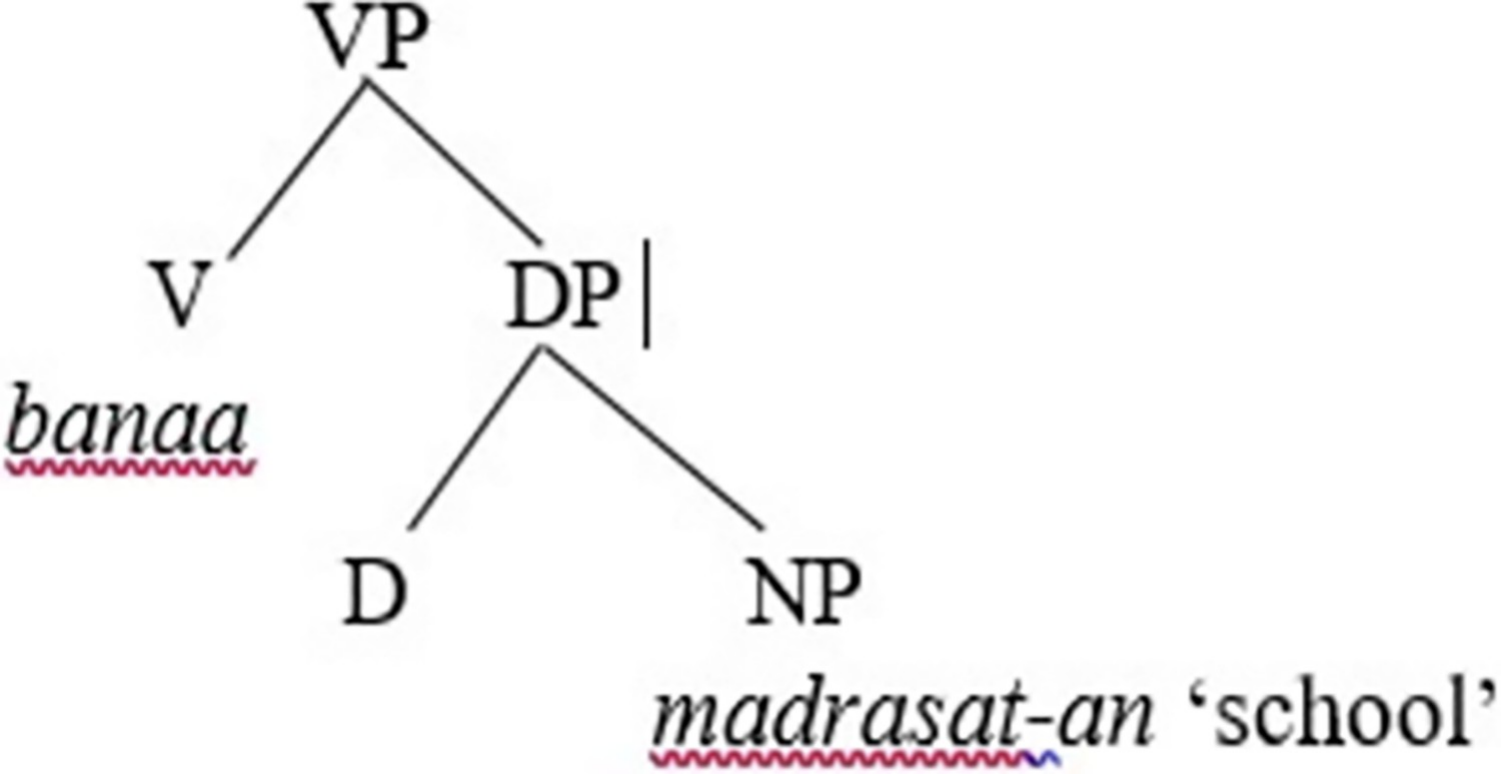
Merge of the lexical V with the object DP in SA.

The lexical V *banaa* ‘built’ merges with the object DP *madrasat-an* ‘school’ to create the VP *banaa madrasat-an* ‘built a school,’ as shown in Fig 3.

Furthermore, the whole VP *banaa madrasat-an* ‘built a school’ merges with the head light verb v to form v-bar. As the syntactic derivation proceeds successfully, agreement between constituents must be ensured; the light head v (acting as the probe) agrees with the object NP in a probe-goal relation. That is, the head v *banaa* ‘built’ is the probe, and the object *madrasat-an* ‘school’ is the goal, the latter (the object) is inside the c-command domain of the former (the verb). As the agreement and probe-goal relations are achieved, the uninterpretable features need to be checked and valued in the syntax. That is, the uninterpretable accusative case feature on the object is valued and deleted and the object DP is also assigned a theta role of ‘Theme.’ Moreover, the v-bar proceeds further and merges with the subject DP *al-rajul-u* ‘the man’; the definite article *al* ‘the’ is merged with the head noun *rajulu* ‘man’ to form *al-rajul-u* ‘the man.’

**Fig 4:**
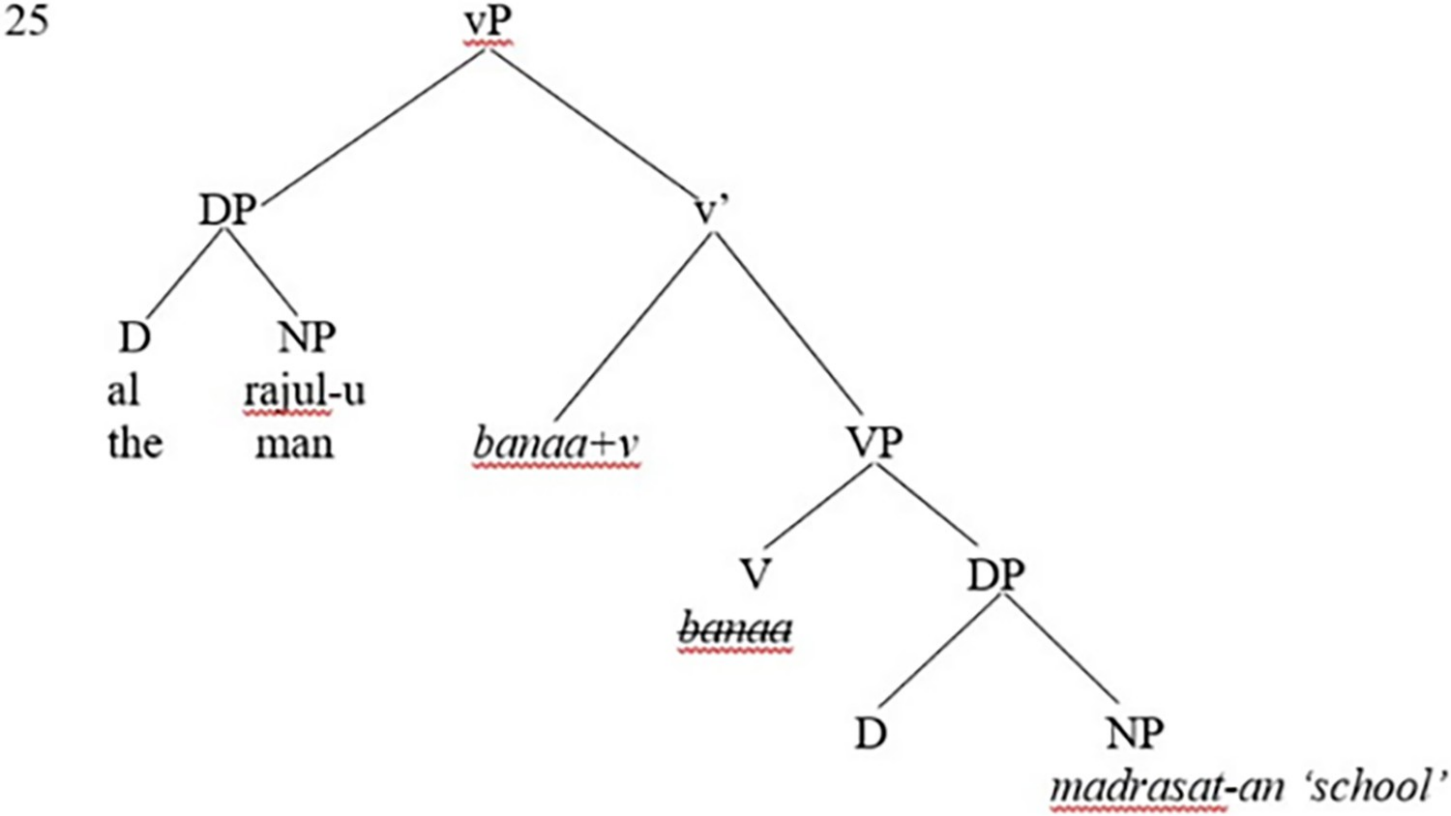
Creation of the inner phase of the vP projection in SA.

Moreover, the syntactic derivation of the construction proceeds further, in which the subject NP (with interpretable phi-features: Third, Singular, Masc., and Definite) enters the derivation, where this Merge operation creates the vP projection; this is the inner phase. Consequently, the head v assigns a theta role of AGENT to the subject DP *al-rajul-u* ‘the man.’ This can be demonstrated in Fig 4.

**Fig 5:**
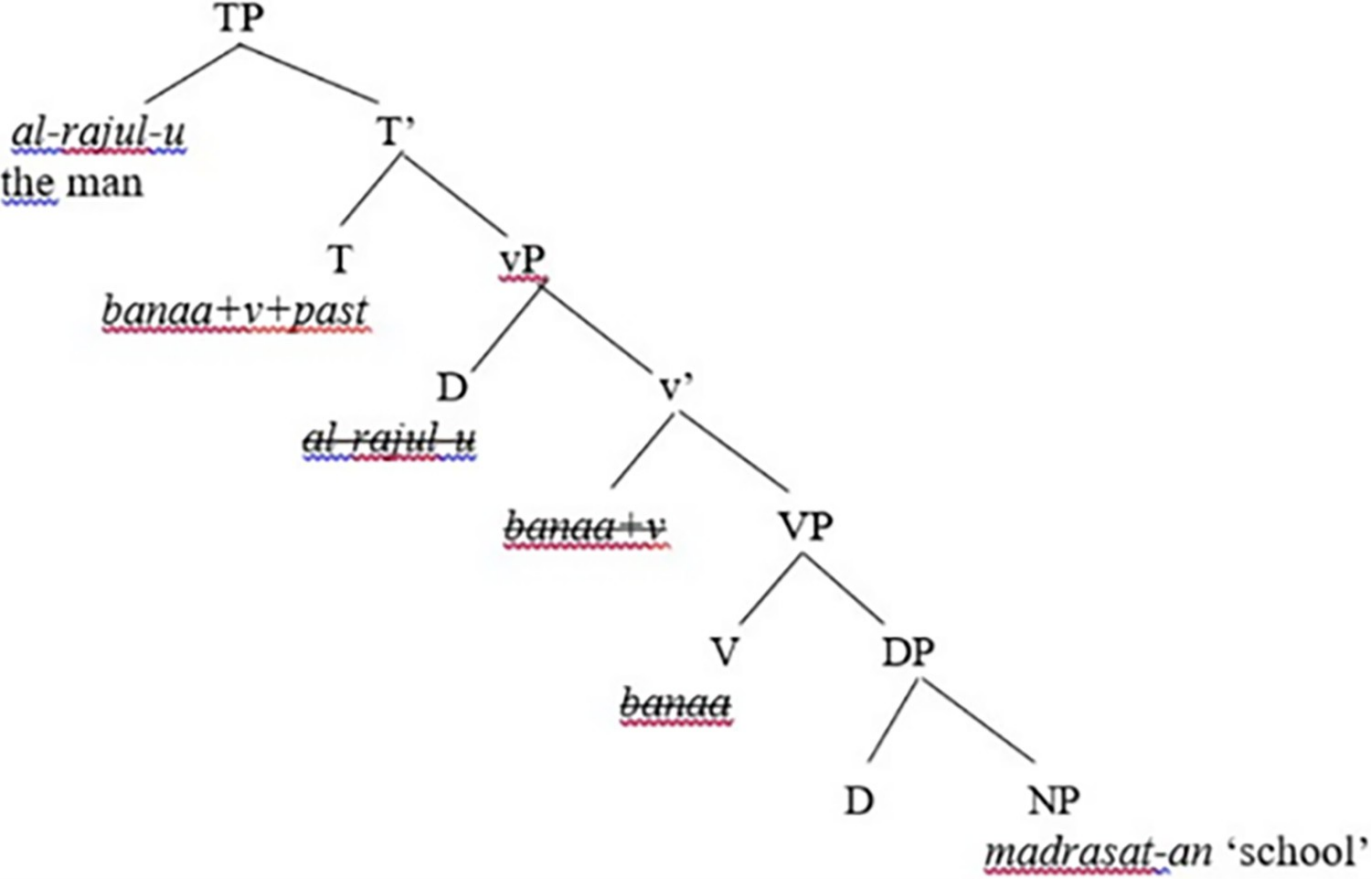
Creation of the TP projection in SA.

As the derivation proceeds further, the whole vP phase merges with the functional head T of TP; it is the head T that shows agreement with the subject DP *al-rajul-u* ‘the man’. The subject NP has an unvalued nominative Case feature that needs to be valued. It is the head T that values the unvalued nominative Case feature of the subject, while the subject DP does value the uninterpretable Φ-features of the head T. That is, the head T agrees with the subject DP *al-rajul-u* ‘the man’, c-commanded by T. The subject is the closest DP to T. Given the EPP feature on the head T, the subject undergoes overt syntactic movement to [Spec, TP], as this is attributed to the strong [EPP] on T, thus creating the TP projection. Besides, since the verb in SA has a rich agreement morphology, it moves to the head T in the overt syntax for feature valuation purposes, as shown in Fig 5.

**Fig 6:**
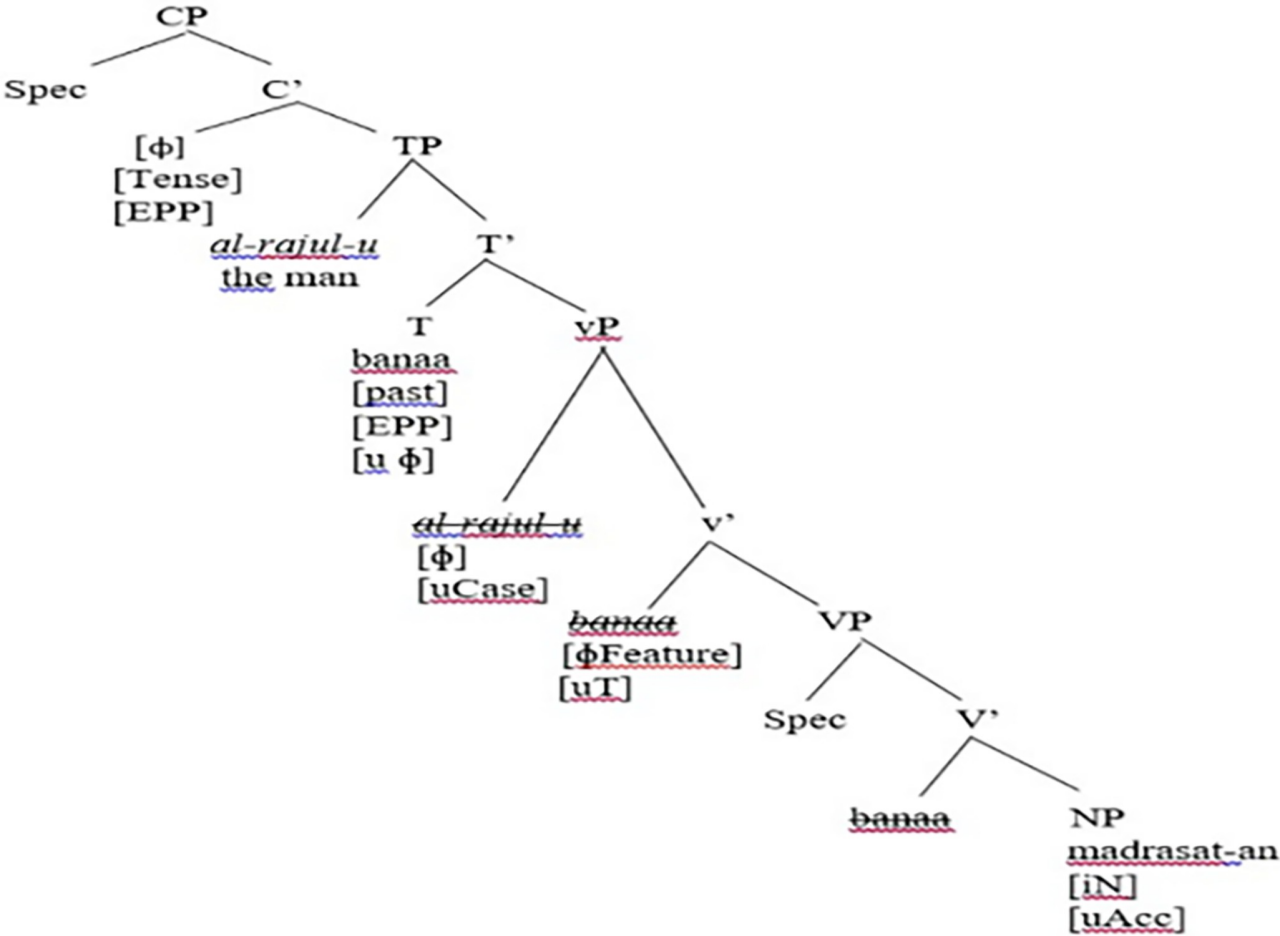
The TP projection merges with C to create the CP projection.

According to Chomsky’s [1, 22] Phase Theory, the whole TP projection merges with the functional head C to create the CP projection; this is the outer phase. A closer look at the vP phase reveals that it has a thematic external argument, namely, the subject DP *al-rajul-u* ‘the man’ and it is considered a phase by itself. Furthermore, the complement DP of the v*P phase is transferred to the LF and PF interface as soon as the head of the higher phase enters the syntactic derivation. Once all features are checked and valued, they get deleted in the syntax; they are transferred to the PF and LF interface. In addition, lower copies such as *banaa* and *al-rajulu* observed in vP and VP are erased and, consequently, the CP configuration is transferred to PF and LF levels. This can be demonstrated in Fig 6.

**Fig 7:**
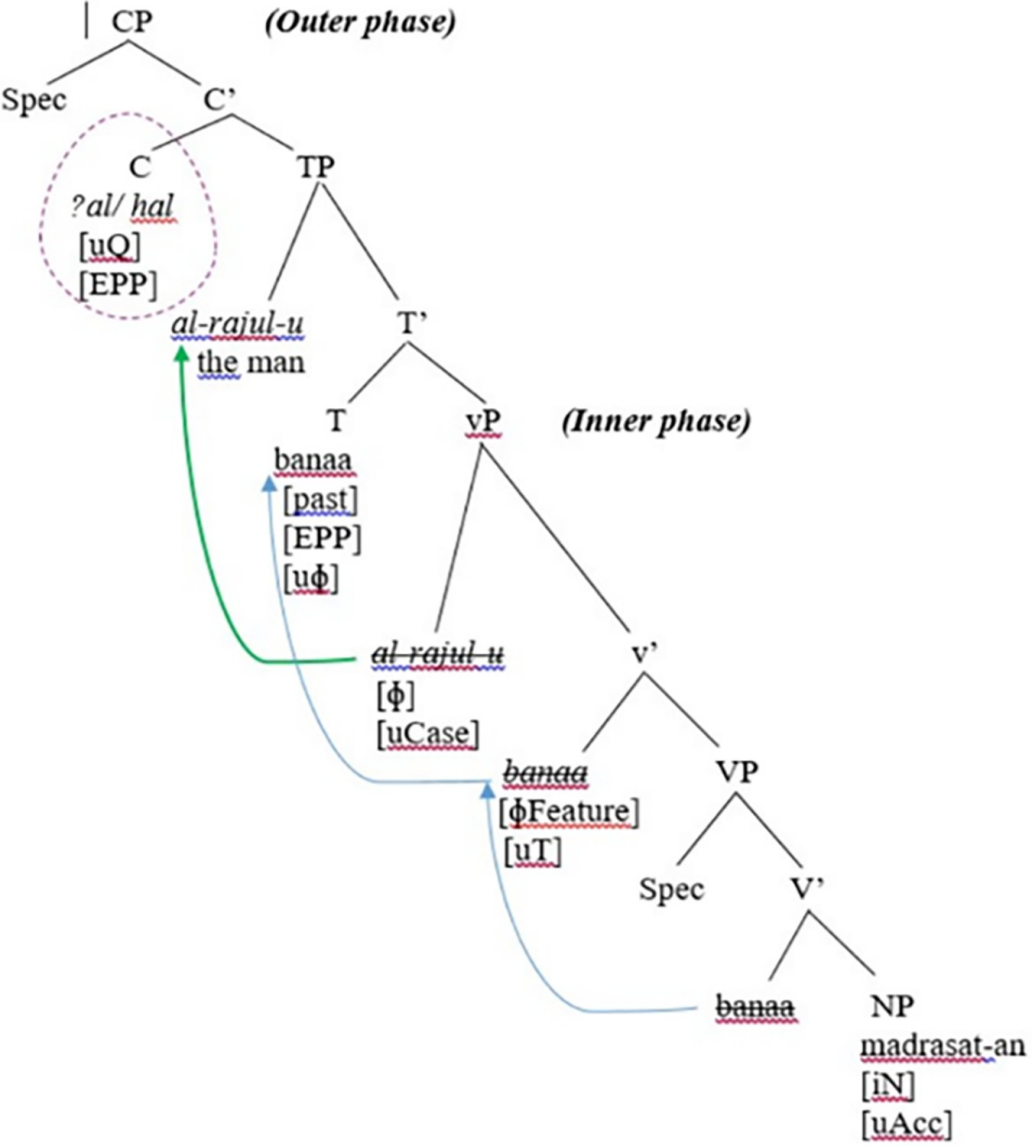
Creation of the CP projection (the outer phase) and feature valuation.

In the preceding analysis, it has been shown that the SVO word order in SA is derived via the movement of the verb to the head T and movement of the thematic subject DP to [Spec, TP], motivated by the EPP feature on the head T. Now, the questions are: How is a yes-no question in SA derived and represented? Do *ʔa* and *hal* undergo syntactic movement or are they base-generated in [Spec, CP]? A closer look at the example in (21b) above represented in the clause structure in Fig 7 below reveals that the question particles *ʔa* and *hal* do not undergo any syntactic movement and they do not belong to the verb tense of the sentence. They are merely morphological affixes: *ʔa* is a bound morpheme, while *hal* is a free morpheme; they are used as a morpho-syntactic device to mark interrogativity in SA grammar.

Moreover, Chomsky [4] proposed that syntactic operations are highly motivated by morphological features. This can explain that syntactic movement (whether in overt or covert syntax) must be initiated to satisfy certain morphological requirements. Given this, Chomsky [4] argued that the functional head C (of CP) carries an operator feature responsible for triggering the syntactic movement of tensed auxiliary verbs and wh-phrases. Chomsky indicated that for an appropriate functional C, the operators move higher for feature licensing to the checking domain of C, namely [Spec, CP]. The minimalist assumption is that all human languages carry a (Q)uestion feature accommodated in the functional head C. However, languages have different syntactic behaviors with regard to the strength/weakness of the Q-feature of the head C.

With the advent of Phase Theory, Chomsky [1, 22] refined the assumptions of the MP, where he proposed that all syntactic operations have to take place before Spell-Out. Chomsky adopted a different position, where he stated that the syntactic operations of wh-movement can be derived as follows: “the wh-phrase has an uninterpretable feature [wh-] and an interpretable feature [Q], which matches the uninterpretable probe [Q] of a complementizer” (p.44). This entails that the uninterpretable Q-feature on the head C operates as a probe that searches for a matching feature of a local goal [wh-] in the search domain. As the probe finds the matching goal, the uninterpretable features located on the probe (Q-feature) and the goal (wh-feature) are valued and then deleted via the Agree operation. Chomsky emphasized that Agree takes place between the probe and the goal, and this entails that the goal raises higher up to the specifier of the probe. Chomsky [22] suggested that the functional head C has an EPP feature held responsible for triggering the movement of auxiliary verbs to the head C or wh-phrases to the Spec of CP.

Given the preceding line of analysis, the authors assume that the Q-feature in SA interrogative structures is strong (i.e., it has some semantic content), and that it is satisfied by “a feature FQ”. The reason why the strong Q of SA cannot undergo Move or Adjunction can be attributed to the lack of auxiliary inversion in the language. Adjunction operation requires movement from the head T to the head C for feature valuation- and this syntactic requirement does not exist in SA. As there is no movement in SA yes-no question derivation, SA resorts to Merge, whereby the Merge operation deletes the strong feature of Q on the head C and in turn renders the structure fully interpretable—since all features are checked and deleted in the course of derivation.

Given that the question particles in SA do not undergo syntactic movement, the authors assume that *ʔa* and *hal* are base-generated in the head C of CP. The head C enters the derivation with an uninterpretable [Q] feature. The authors assume that the head C carries the Question feature [Q], which has an uninterpretable [Q] feature. Besides, the head C is endowed with an EPP feature. *ʔa* and *hal* have interpretable Q features that must be checked against the uninterpretable [Q] feature on the head C; the latter (the head C) acts as the phase head of CP; the outer phase. The authors assume that phase head C has an EPP feature that is responsible for checking and valuing the interpretable features of *ʔa* and *hal* in the base-generated position of C. Once the interpretable Q features of *ʔa* and *hal* are checked (and valued) against the uninterpretable [Q] feature on the head C, they are deleted via the Agree operation. When agreement takes place between the head C and *ʔa/ hal*, all uninterpretable features on the probe C and on the goal *ʔa*/ *hal* are valued and deleted, thus a yes-no question is derived. Consequently, the CP configuration is transferred to the PF and LF interface. This can be illustrated in Fig 7.

### Derivation of yes-no questions in VSO structures

**Fig 8:**
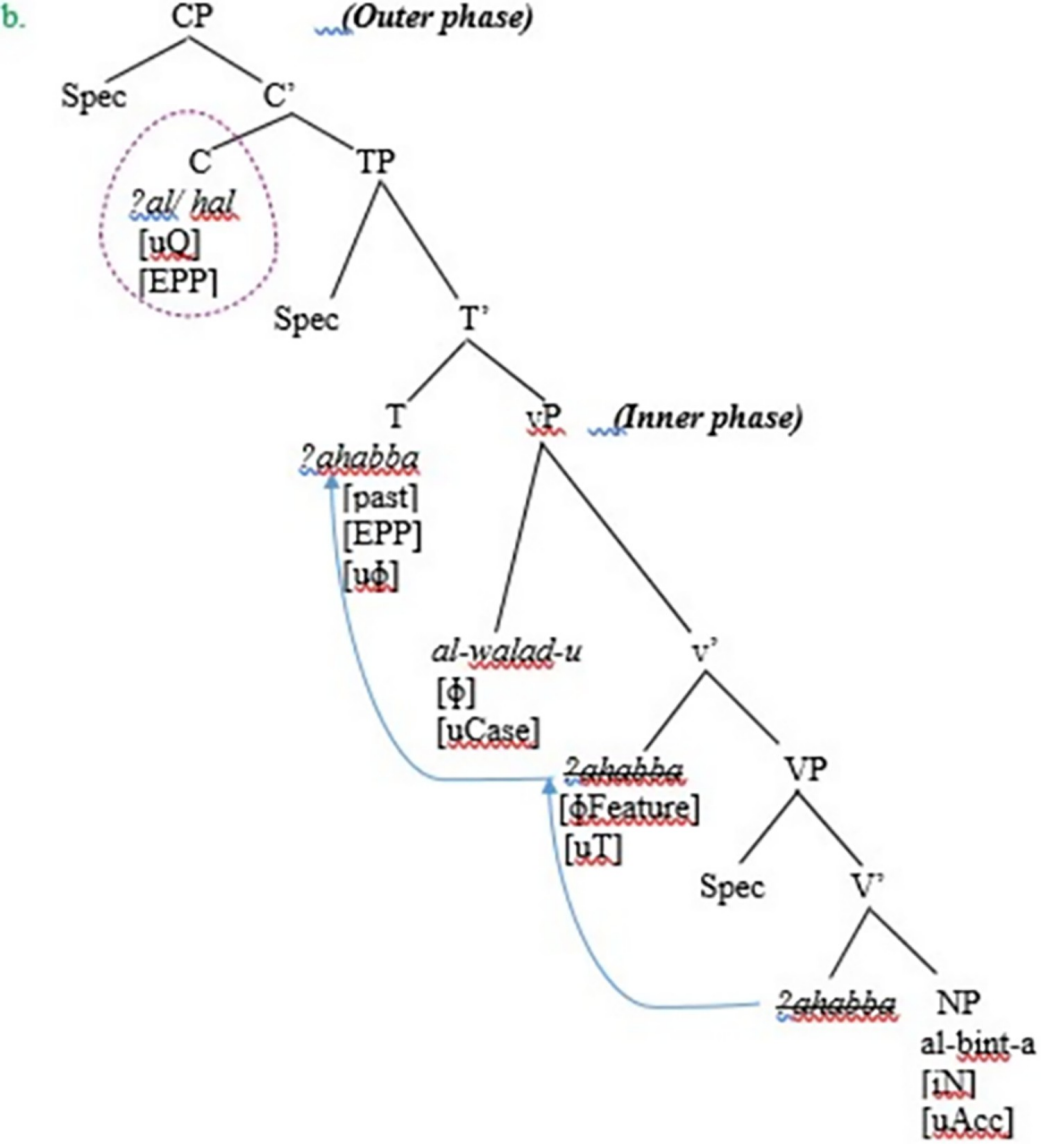
Derivation of yes-no questions in VSO word order in SA.

In this sub-section, the authors explore how a yes-no question is derived and represented in the VSO word order in SA. Let us consider the following example in (22) and how it is represented on a clause structure of SA in Fig 8 to illustrate the point.

22a **ʔa/ hal**? ahabba al-walad-u al-bint-a

 **Q** loved the-boy-nom the-girl-acc

 ‘Did the boy love the girl?’.

The syntactic derivation of yes-no questions in VSO proceeds further as follows in (22). The lexical verb *ʔahabba* ‘loved’ merges with the object complement DP *al-bint-a* ‘girl’ to form the VP *ʔahabba al-bint-a* ‘loved the girl.’ Next, the VP *ʔahabba al-bint-a* ‘loved the girl’ merges with the light v to create a v-bar. Furthermore, the light v acts as the probe and agrees with the object NP via a probe-goal relation. It is this light v which is the head phase. The object is located inside the c-command domain of the verb. Since agreement and probe-goal relations are ensured, the uninterpretable features must be checked and valued; the uninterpretable accusative case feature on the object DP *al-bint-a* ‘girl’ is valued and deleted; the object DP is also assigned a PATIENT/THEME theta role. Besides, the v-bar merges with the subject DP *al-walad-u* ‘the boy’ to create the vP projection. Furthermore, as the derivation of the interrogative structure proceeds, the subject DP, which has interpretable phi-features, enters the syntactic derivation, where the Merge operation creates the vP phase; the inner phase.

Moreover, unlike SVO, the subject DP in the VSO order in SA does not move to [Spec, CP] in the overt syntax for feature valuation considerations, for the reason that the head T lacks an EPP feature responsible for triggering the overt movement of the subject DP; rather, the subject stays in-situ in [Spec, vP]. The vP phase merges with the head T of TP. The head T must agree with the subject DP *al-walad-u* ‘the boy; where the unvalued nominative Case feature of the subject DP is checked and valued by the head T at LF. The reason why the subject nominative case feature is valued at LF can be attributed to the fact that the head T lacks the EPP feature, which is supposed to trigger movement of the subject DP to [Spec, TP]. Hence, the subject DP remains in-situ inside [Spec, vP]. Given the assumptions of Phase Theory, the head T acts as the probe, which searches for a local goal (the subject) with which it agrees and assigns a nominative case at LF. Although the subject is the closest DP to the head T, it cannot undergo movement to [Spec, TP], because T does not have the EPP feature. Furthermore, because T is strong and affixal in SA, it attracts the complex V+v to move to T to check and value the tense feature and provide a host for it. Besides, since the verb in SA has a rich agreement system, it moves to T in the overt syntax for feature valuation requirements. It can, however, be noted that TP is not a phase.

On the other hand, the authors have already assumed that *ʔa* and *hal* are base-generated in the head C of CP. What applies to the derivation of yes-no questions in SVO also applies to the same in VSO. The phase head C bears the [Q]uestion feature, which has an uninterpretable Q- feature; C enters the derivation with an uninterpretable Q-feature. Phase head C has an EPP feature, which is responsible for feature valuation. Furthermore, *ʔa* and *hal* have interpretable Q features that must be checked against the uninterpretable Q-feature on the functional head C; the latter (C) acts as the phase head of the CP phase, which Chomsky terms as ‘the outer phase.’ It can be assumed that the phase head C has an EPP feature, which triggers valuing the interpretable features of *ʔa* and *hal* in their base-generated position of C. As soon as the interpretable Q features of *ʔa* and *hal* are valued against the uninterpretable Q-feature on C, they are deleted. Once Agree operation takes place between the probe C and the goal *ʔa/hal*, all uninterpretable features on the probe C and the goal *ʔa*/*hal* are valued and deleted, hence an acceptable derivation of a yes-no question is created. Moreover, when all syntactic operations have been completed in the CP/vP phase, the complement of the phase head becomes impenetrable to further syntactic operations. It can be observed that the syntactic analysis of the question particles in SA obeys Chomsky’s [22, 30] Phase Impenetrability Condition (PIC). The present analysis is in line with that of Chomsky; this explains that the reason why the complement NP or the domain of the phase becomes impenetrable can be attributed to the fact the domain undergoes transfer simultaneously to the PF component and the semantic level is assigned the appropriate representations.

Let us examine how the question particles *ʔa* and *hal* can be used in the passive voice in the derivation of yes-no interrogatives in VSO word order. This can be demonstrated in (23) and (24).

23a. kataba mohammed-un al-risa:lat-a   **(active voice, VSO)**

 wrote Mohammed-nom the-letter-acc

  ‘Mohammed wrote the latter.’

b. **hal/ʔa-** kataba mohammed-un al-risa:lat-a   **(active voice, VSO)**

 Q wrote Mohammed-nom the-letter-acc

  ‘Did Mohammed write the letter?’

c. *kataba **hal/ʔa-** mohammed-un al-risa:lat-a

 wrote Q Mohammed-nom the-letter-acc

24a. **kutibat** al-risa:lat-u   **(passive voice, VSO)**

  be-written the-letter-no

   ‘Mohammed wrote the latter.’

b. **hal/ʔa-** kutibat al-risa:lat-u   **(passive voice, VSO)**

  Q be-written the-letter-no

  ‘Was the letter written?’

c. *kutibat **hal/ʔa-** al-risa:lat-u

  be-written **Q**  the-letter-no

(23a) is an active sentence, while (23b) is a yes-no question in VSO word order in SA. (23c) is ungrammatical because Standard Arabic does not allow this odd interrogative construction. The ungrammaticality of (23c) can be attributed to the fact that the question particles cannot be used between the verb and the subject. That is, the question particle must be placed in the initial (but not in the medial or final) position of a yes-no interrogative.

On the other hand, (24a) is a passive sentence, while (24b) is a yes-no question in VSO order. (24b) shows that even in the passive voice the question particle must begin a yes-no question in Standard Arabic. The ungrammaticality of (24c) can be explained as that of (23c); the interrogative particle must be used clause-initially. It can be concluded that in the passive and active yes-no constructions the question particle must be placed in the beginning of the interrogative.

Moreover, let us illustarte in the following examples in (25) how the question particles can be used in yes-no interrogatives, which have a complementizer acting as an island.

25a. qa:la zayd-un ʔanna al-tulla:b-a ghadar-u:

 said Zayd.nom that the-students.acc left.3m.pl.nom

   ʻZayd said that the students left.ʻ

b. **hal/ʔa-** qa:la zayd-un   **ʔanna** al-tulla:b-a ghadar-u:?

 **Q** said Zayd.nom that the-students.acc left.3m.pl.nom

 ʻDid Zayd say that the students left?ʻ

c. *qa:la zayd-un   **ʔanna hal/ʔa-** al-tulla:b-a ghadar-u:

  said Zayd.nom that   **Q** the-students.acc left.3m.pl.nom

A closer look at the example in (25b) reveals that the question particles *hal* and *ʔa* can be used in the initial position of a yes-no question with a complementizer clause. (25b) is an example of yes-no question that have a comlementizer. However, (25c) illustrates that SA does not permit the derivation of a yes-no interrogative, where the question particle is not placed clause-initially, and this explains the ungrammaticality of (25c). Since, the question particle is a morphological affix and does not undergo any synatctic movement in the derivation of a yes-no interrogative, the presence or absence of the complementizer *ʔinna* ʻthatʻ plays no role in the analysis of these question particles; these question particles are merely intererogative morphemes used to mark interrogativity.

## Conclusion

The study has answered the research questions in a systematic fashion and examined the interaction between the SA data on question particles and Chomsky’s [1, 22] Phase Theory, which revealed interesting findings. The analysis revealed that, unlike English, the SA question particles *ʔa* and *hal* are not part of the verb morphology and they are found to be merely morphological affixes placed clause-initially to form interrogativity. Besides, *ʔa* and *hal* do not show any agreement with verbs and their subject DPs; they have nothing to do with agreement inflection as they do not inflect for tense or Case. Furthermore, *ʔa* is used as a bound morpheme, while *hal* is a free morpheme. What is interesting about the difference between *ʔa* and *hal* is that the former (*ʔa*) can be used with both affirmative and negative yes-no questions, while the latter (*hal*) is only used with affirmative interrogatives. The reason behind the difference can be attributed to the fact in the derivation of a well-formed construction of a negative yes-no question, SA resorts to banning the occurrence of two free morphemes (i.e., *hal + negative marker) in an interrogative, where one free morpheme cannot follow the other. It only allows a bound morpheme (i.e., *ʔa* + a negative free marker) to co-occur with a free negative marker in the same interrogative. Hence, it is concluded that the syntactic representation *hal + neg … is banned, while that of *ʔa* + neg … is permitted in SA grammar.

Moreover, as *ʔa* and *hal* do not undergo any syntactic movement to the [Spec, CP] projection while deriving a yes-no question; it is assumed that *ʔa* and *hal* are base-generated in the head C of [Spec, CP] in SVO and VSO structures, where C enters the derivation with an uninterpretable [Q] feature. It is also assumed that C (which acts as the phase head) is endowed with an EPP feature responsible for feature evaluation of *ʔa* and *hal* in the base-generated position of C. The interpretable Q features of *ʔa* and *hal* are checked against the uninterpretable [Q] feature on C, being the phase head of CP, the outer phase. As soon as Q features of *ʔa* and *hal* are valued against the uninterpretable [Q] feature on C, they are deleted in the syntax. In other words, once Agree operation takes place between probe C and the goal *ʔa*/*hal*, all uninterpretable features on probe C and the goal *ʔa*/*hal* are valued and deleted, hence yielding a well-formed yes-no question. Consequently, the CP configuration is transferred to the PF and LF interface for appropriate interpretation. Furthermore, the preceding analysis of SA question particles showed that once all syntactic operations have been completed in the CP phase, the complement of the phase head becomes impenetrable to further syntactic operations.

In addition, the study provided some theoretical implications. The findings of the study revealed further support for the applicability of Chomsky’s Phase Theory in the analysis of the SA question particles. The analysis demonstrated that SA obeys the Phase Impenetrability Condition of Chomsky [22, 30], which states that when all syntactic operations in a given phase have been achieved, the domain of that phase becomes impenetrable to any syntactic operation in the syntax. Besides, the present analysis is in line with that of Chomsky [1]; it explained that the reason why the complement NP/ or the domain of the phase in SA data becomes impenetrable to syntactic operations can be attributed to the fact the domain undergoes transfer simultaneously to the PF interface and the semantic component is assigned the proper representation in the syntax. It is hoped that analysis of the SA question particles will contribute to the understanding of the existing literature on the morpho-syntax of question particles not only in the Semitic language family but also in other languages of the world, particularly those languages which have clause-initial question particles.
